# Longitudinal *in vitro* surveillance of *Plasmodium falciparum* sensitivity to common anti-malarials in Thailand between 1994 and 2010

**DOI:** 10.1186/1475-2875-11-290

**Published:** 2012-08-21

**Authors:** Daniel Parker, Rujira Lerdprom, Wanna Srisatjarak, Guiyun Yan, Jetsumon Sattabongkot, James Wood, Jeeraphat Sirichaisinthop, Liwang Cui

**Affiliations:** 1 Department of Anthropology, The Pennsylvania State University, 409 Carpenter Building, University Park, PA, 16802, USA; 2 Population Research Institute, The Pennsylvania State University, 601 Oswald Tower, University Park, PA, 16802, USA; 3 Vector Borne Disease Training Center, Pra Budhabat, Saraburi, 18120, Thailand; 4 Public Health, University of California, Irvine, CA, 92697, USA; 5 Faculty of Tropical Medicine, Mahidol University, Bangkok, 10400, Thailand; 6 Department of Entomology, The Pennsylvania State University, 501 ASI Building, University Park, PA, 16801, USA

**Keywords:** *Plasmodium falciparum*, Drug resistance, Artemisinin, Mefloquine, Thailand, The Greater Mekong subregion

## Abstract

**Background:**

Drug and multidrug-resistant *Plasmodium falciparum* malaria has existed in Thailand for several decades. Furthermore, Thailand serves as a sentinel for drug-resistant malaria within the Greater Mekong sub-region. However, the drug resistance situation is highly dynamic, changing quickly over time. Here parasite *in vitro* drug sensitivity is reported for artemisinin derivatives, mefloquine, chloroquine and quinine, across Thailand.

**Methods:**

Blood was drawn from patients infected with *P. falciparum* in seven sentinel provinces along Thai international borders with Cambodia, Myanmar, Laos, and Malaysia. *In vitro* parasite sensitivity was tested using the World Health Organization’s microtest (mark III) (between 1994 and 2002) and the histidine-rich protein-2 (HRP2)-based enzyme-linked immunosorbent assay (in 2010). Following World Health Organization protocol, at least 30 isolates were collected for each province and year represented in this study. Where possible, *t*-tests were used to test for significant differences.

**Results:**

There appears to be little variation across study sites with regard to parasite sensitivity to chloroquine. Quinine resistance appears to have been rising prior to 1997, but has subsequently decreased. Mefloquine sensitivity appears high across the provinces, especially along the north-western border with Myanmar and the eastern border with Cambodia. Finally, the data suggest that parasite sensitivity to artemisinin and its derivatives is significantly higher in provinces along the north-western border with Myanmar.

**Conclusions:**

Parasite sensitivity to anti-malarials in Thailand is highly variable over time and largely mirrors official drug use policy. The findings with regard to reduced sensitivity to artemisinin derivatives are supported by recent reports of reduced parasite clearance associated with artemisinin. This trend is alarming since artemisinin is considered the last defence against malaria. Continued surveillance in Thailand, along with increased collaboration and surveillance across the entire Greater Mekong sub-region, is clearly warranted.

## Background

The anti-malarial drug use history and drug resistant situation in Thailand largely mirror that of the Greater Mekong sub-region (GMS), which consists of Cambodia, Vietnam, Laos, Thailand, Myanmar, and Yunnan Province of China. Drug resistance in malaria has plagued this region for decades [1-3]. Resistance to chloroquine (CQ) emerged in the late 1950s and early 1960s. In the early 1970s sulphadoxine and pyrimethamine (S/P) became the primary anti-malarial drug for *Plasmodium falciparum* infection in Thailand; however, within a decade the combination was largely ineffective. The focus then turned back to quinine (QN); however because of apparent resistance, it needed to be reinforced with tetracycline. By 1985 mefloquine (MQ), in combination with S/P, became the first-line drug for confirmed *P. falciparum* infection and by 1991 MQ was being used alone. However, by the late 1980s MQ was already losing its efficacy, especially along the Thailand-Cambodia and Thailand-Myanmar borders [3]. In 1993 Thailand began some small trials using artesunate (ATS) in these areas and in 1995 ATS-MQ combination became the official standard therapy for confirmed *P. falciparum* cases in regions known to have high levels of MDR malaria (specifically along the Thai-Myanmar and Thai-Cambodian borders) [3]. To date, artemisinin (ART)-based combination therapies have been adopted as the front-line drugs in most *P. falciparum*-endemic areas of the world because of the global problem of multidrug resistance [4]. In the last several years potential resistance to ART derivatives, manifested as delayed parasite clearance, has been reported along the Thailand’s border regions with Cambodia and Myanmar [5-9]. To control the potential spread of the resistant parasites, surveillance efforts are heightened and a containment plan is being deployed in this region.

The GMS has historically been a breeding ground for drug and multidrug resistant (MDR) malaria parasites. The reasons for emergence of drug resistance in this region are not entirely clear. One potential contributor to the problem is loose regulation of anti-malarials in many of the nations within the GMS, where large proportions of the anti-malarial drugs are counterfeit or substandard [10]. Another may be misuse of anti-malarial drugs and poor compliance of patients to drug regimens [11]. All these factors can lead to sub-therapeutic levels of drug residues in the blood, a condition that favours the selection for drug resistant parasites. In addition, human migration has played a major role in mediating rapid spread of the resistant parasites both across the GMS and to other regions of the world [12]. For example, there was a suggestion that MDR strains originated along the Thai-Cambodian border and then moved, along with migrant workers, to the Thai-Myanmar border [3]. When MDR strains were first recognized in north-western Thailand in early 1990s, there was a high level of migration from the south-east to the north-west, a pattern that seems consistent with this suggestion. Regardless of the origins of drug resistance, surveillance systems that monitor the drug resistance situation in the GMS are imperative for malaria control efforts in this region.

In Thailand, anti-malarial drug resistance has been monitored over time at several sentinel sites. This paper presents longitudinal surveillance data from *in vitro* tests of drug sensitivity to four commonly used anti-malarial drugs (ART, CQ, QN, and MQ) between 1994 and 2010 from several sentinel provinces along Thai borders. Previous studies have noted abnormally high effective concentration values that inhibit 50% of the parasite growth (IC50) for MQ in north-western provinces, however resistance was thought to have plateaued around the turn of the century [3]. MQ resistance, at least *in vitro*, subsequently rose to much higher levels. Furthermore, this investigation pointed to potentially increasing resistance to ART and its derivatives. As the last line of defence against MDR malaria parasites, this finding is alarming and it underlines the importance of continued drug resistance surveillance.

## Methods

### Drug resistance monitoring sites

**Figure 1:**
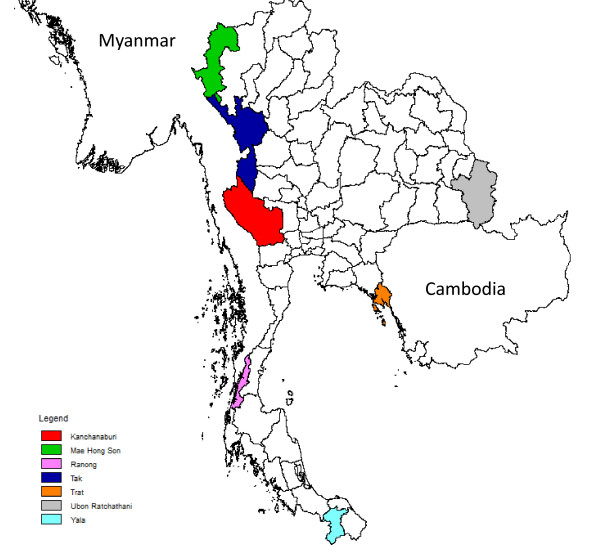
**Drug resistance sentinel sites along Thai borders.** Colour scheme matches that of Figure 2.

Longitudinal *in vitro* monitoring of drug sensitivity of *P. falciparum* clinical isolates was conducted in Trat province bordering Cambodia, Ranong, Mae Hong Son, Kanchanaburi and Tak provinces along Thai-Myanmar border, Yala province in the South, and Ubon Ratchathani province bordering Laos (Figure 1). These sentinel sites have been strategically selected because they historically have been among the most malarious provinces and border the malarious regions in neighbouring countries.

### Patients and parasite samples

Drug resistance monitoring was conducted at government-run malaria clinics in the sentinel provinces, which offer free diagnosis and treatment to malaria patients. Adult patients with uncomplicated *P. falciparum* malaria were recruited to this study after giving written consent. This study was conducted under the auspices of the Bureau of Vector Borne Diseases, Ministry of Public Health, Thailand. Infection by *P. falciparum* was diagnosed based on microscopic examinations of Giemsa-stained thick and thin blood smears, and only those samples with >2,000 asexual parasites/μl of blood were used for *in vitro* drug assays. After verifying that the patients had not taken anti-malarial drugs or antibiotics within the preceding two weeks, 1 ml of venous blood was drawn for *in vitro* drug assay. In each province in a given year, at least 30 clinical blood samples were obtained for anti-malarial drug assays. Isolates with known mixed-species infections were excluded from this study.

### *In vitro* drug sensitivity assays

*In vitro* drug sensitivity of fresh *P. falciparum* isolates has been monitored with regards to four anti-malarial drugs: ART, CQ, QN, and MQ. Between 1993 and 2002, the World Health Organization microtest (mark III) was used to test drug sensitivity [13]. The microtest requires a parasitaemia of 1,000–80,000 parasites/μl blood. Microtiter plates were pre-dosed with serially diluted anti-malarial compounds and a control well (without drug) was included for each sample. Blood from patients was mixed with RPMI 1640, mixed with complete medium, and dispensed into the wells of the plates at 50 μl/well. Plates were incubated at 37°C for 24–36 h in a candle jar and thin smears were examined. Only samples with >20% parasite matured to schizonts in the control wells were used for data analysis.

*In vitro* testing was halted from 2003 – 2009 because of reduced funding. In 2010, testing switched to the comparatively less labour-intensive histidine-rich protein-2 (HRP2)-based enzyme-linked immunosorbent assay (ELISA) [14]. This method requires a parasitaemia of >500 parasites/μl of blood, making it easier to enrol patients in parasite drug sensitivity studies. As with the mark III method, plates were pre-dosed with anti-malarial compounds. IC50 values were calculated for each parasite isolate and each drug using regression analysis. The commercial HRP2 assay came from Cellabs (http://cellabs.com.au/) and pre-dosed plates were obtained from Immunology Consultants Laboratory, Inc. (http://www.icllab.com/).

### Statistical analysis

Data from individual assays for the microtest III method were discarded after summary statistics were calculated, making quality assurance and statistical analysis difficult or impossible for these data. Data from the HRP2 method are preserved in digital format and will be retained so that future analyses can be done on variance between assays within and across laboratory sites as well as statistical analysis on IC50 dynamics. The HRP2 data were analysed using multiple *t-*tests with Bonferroni correction.

## Results

**Figure 2:**
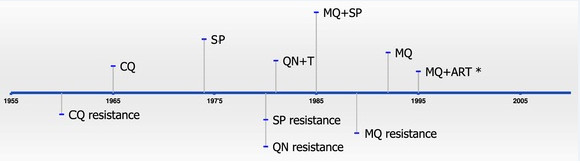
**Timeline showing the official use of anti-malarials (top) and anti-malarial resistance (bottom) in Thailand.** In some cases, anti-malarials were in use prior to becoming the official anti-malarial and, therefore, drug resistance existed prior to official use (e.g., CQ). CQ = chloroquine; SP = sulphadoxine-pyrimethamine; QNN = quinine; QNN + T = quinine + tetracycline; MQ = mefloquine; ART = artemisinin. *MQ + ART began being used as a standard therapy for *Plasmodium falciparum* in Tak Province and the south-eastern border with Cambodia (Trat Province).

The national drug policy changes in Thailand have been made based on clinical observations, parasitaemia levels after administering anti-malarials (regardless of febrile status), on the efficacy of anti-malarial drugs (Figure 2).

### Chloroquine

**Figure 3:**
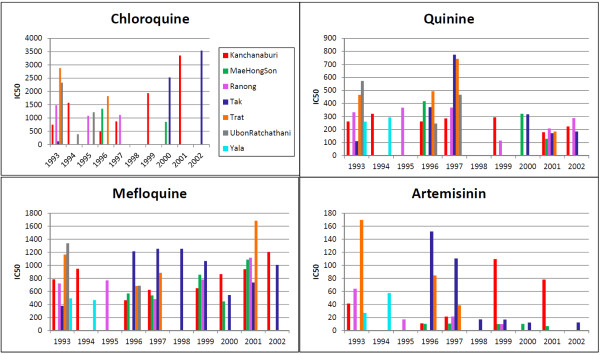
**Results from the microtest (mark III) drug sensitivity tests (1993–2002).** Colour scheme matches that of Map 1. These data have been collected by the Bureau of Vector Borne Diseases, Ministry of Public Health, Thailand and have not previously been reported. For each year and province, at least 30 clinical isolates were used to calculate the average IC50. The detailed results of each assay have since been discarded, meaning that error bars and variance between assays cannot be calculated for this component of the data.

**Figure 4:**
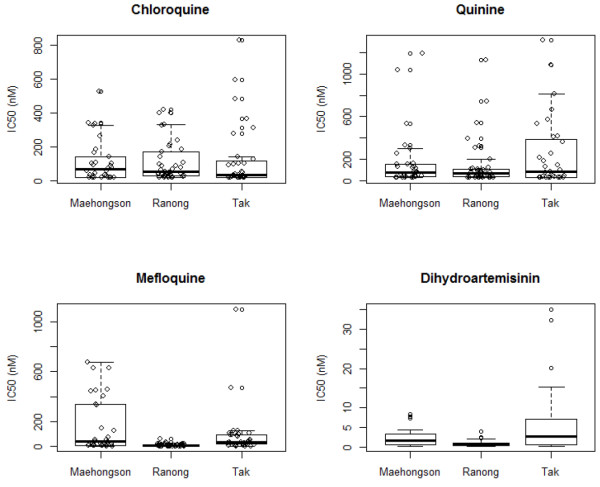
**Results from the HRP2 data (2010).** These data have been collected by the Bureau of Vector Borne Diseases, Ministry of Public Health, Thailand and have not previously been reported. Box plots represent the mean IC_50_ as well as the quartiles and 95% confidence intervals of the data.

When CQ became the official anti-malarial drug used in Thailand around 1965, resistance to this drug already existed. CQ use for *P. falciparum* infections has long since been halted. These data showed that CQ IC50 values varied greatly between some provinces. In many regions such as the eastern provinces, there was a trend of decrease in CQ resistance (Figure 3). In contrast, CQ IC50 values remained high or even increased in the west (e.g., Kanchanaburi and Tak provinces) around the turn of the century. Unfortunately, CQ sensitivity monitoring only continued in the three western provinces bordering Myanmar (Figure 4). In each of the provinces, the year 2010 CQ IC50 values from the HRP2 method showed a wide range of variability. However, there were no significant differences among the three provinces (*t*-test, *p*-value > 0.05).

### Quinine

Following decreased efficacy of S/P, QN, in combination with tetracycline, became the official anti-malarial in the early 1980s. It was necessary to couple this drug with tetracycline because there was already resistance to QN alone [15]. *In vitro* monitoring of QN sensitivity began in 1993, and by this time MQ had replaced QN. These data showed that before 1997, *in vitro* sensitivity to QN was relatively unchanged in some provinces (Yala, Trat, Ubon Ratchathani, Ranong and Kanchanaburi) or even on the rise in other provinces (Tak and Mae Hong Son). After 1997, there was a decreasing trend in QN IC50 values in all provinces (Figure 4). Similar to the pattern seen in the CQ data, the QN IC50 values derived from the HRP2 method showed great variability (Figure 4), but no significant differences (*t*-test, *p*-value > 0.05) were detected across the three sentinel regions in 2010.

### Mefloquine

MQ has been in use since the mid-1980s and by the early 1990s MQ alone was the official anti-malarial used in Thailand. These data showed that MQ IC50 values remained consistently high (Figure 3), although in some provinces (such as Tak and Mae Hong Son) there was a trend of slight decrease in IC50 values prior to 2000. In the eastern border area (Trat Province), MQ IC50 values remained high and even increased in 2001. In the western border area, Tak Province showed consistently high and potentially rising MQ resistance throughout this time period. Data from Kanchanaburi also showed a steady trend of increase in IC50 values. From the 2010 data using the HRP2 method (Figure 4), MQ IC50 values were significantly higher in Mae Hong Son Province when compared to Ranong (*p*-value = 0.0006; C.I. = 73.88, 240.20). There were several outliers in the data from Tak that appeared much higher than values in either Mae Hong Son or Ranong. However, the combined values from Tak Province did not reach statistical significance when compared to either of the two provinces.

### Artemisinin

ATS use began in some regions of Thailand during the early 1990s and by 1995 ATS-MQ became the official anti-malarial for treating uncomplicated falciparum malaria in selected areas along the Thai-Myanmar and Thai-Cambodian borders. From these data, most of the provinces showed relatively high sensitivity to ART (Figure 3). However, it appeared that ART IC50 values in Trat and Tak provinces were relatively high early on, but subsequently decreased during the 1990’s. In contrast, ART IC50 values in Kanchanaburi were consistently high. Data from the HRP2 method performed in 2010 indicated that *in vitro* IC50 values to dihydroartemisinin (DHA) were significantly higher in Tak Province when compared to both Mae Hong Son (*p*-value = 0.0321; C.I. = 0.34, 7.15) and Ranong (*p*-value = 0.0032; C.I. = 1.89, 8.55) (Figure 4). After applying a Bonferroni correction to control for multiple tests, the difference between Tak and Ranong remained significant.

## Discussion

These data show that the anti-malarial drug resistance situation in Thailand (and most likely the GMS) is dynamic with considerable fluctuation over time. Surveillance systems in the GMS are therefore crucial for malaria control efforts, especially with regard to the use of anti-malarials. Since drug resistant strains appear to have arisen in this area and subsequently spread to other parts of the world in the past, surveillance in this area is important not only for the immediate region but also for the entire world. This is perhaps especially the case with the potential for ART-resistant strains coming out of the GMS. If ART-resistant strains were to spread to Sub-Saharan Africa, the results could be catastrophic.

CQ was widely used globally following its introduction after World War II. In Thailand, as in other parts of the world, resistance quickly developed. By the late 1950s, Thailand already experienced CQ-resistant parasites, even before CQ was designated the official anti-malarial. While CQ use for *P. falciparum* infections was discontinued in Thailand during the 1970s, other parts of the world, especially Africa, continued to use the anti-malarial for most of the 20th century. Resistance to the drug was widespread anywhere it was in use. Tests for *in vitro* and *in vivo* sensitivity to CQ have subsequently shown increased sensitivity shortly after removal of drug pressure [16]. Furthermore, mutations that have been associated with CQ resistance (in PfCRT and Pfmdr1) have been shown to decrease in frequency after cessation of CQ use [16-18]. These data indicate that in Thailand CQ resistance has decreased in eastern provinces while potentially rising in western Provinces (such as Tak and Kanchanaburi). This pattern may in part be explained by the continued use of CQ for treating *Plasmodium vivax* infections. Furthermore, some research has suggested that mixed infections are relatively widespread and under-diagnosed in Thailand [19]. If patients infected with both species of malaria are diagnosed with *P. vivax* and subsequently treated with CQ, this will inevitably retain the CQ pressure and therefore CQ resistance in *P. falciparum*.

Previous research has noted abnormally high MQ IC50 values along the Thai-Myanmar border in the mid-1990s [3]. Other studies in the north-western region of Thailand showed a strong correlation between high MQ IC50 values and clinical failure rates [20]. Decreased parasite sensitivity to MQ corresponded to increased *P. falciparum* gametocytaemia in the study population [20]. However, as ACT was introduced in the area around 1995, it appeared that parasite sensitivity to MQ increased. This increase in parasite sensitivity to MQ has largely been attributed to the introduction of ART and its derivatives for *P. falciparum* infections [20,21]. However, these data indicate that after a decrease in IC50 values around the turn of the century, MQ IC50 values have again begun to rise in the north-western areas of Thailand. There is some evidence that ART resistance may be rising in Tak Province. In addition, at both Thai-Cambodian and Thai-Myanmar border areas, clinical resistance to ATS-MQ has been on the rise [3,6,22]. The fact that sensitivities to MQ and ART family drugs are often correlated [23,24] and ATS-MQ is the most commonly used ACT formulation in this region suggest that MQ sensitivity needs to be continuously monitored.

These results also have broad relevance for other regions where combination therapy is being implemented in order to combat parasite resistance to monotherapies. These data suggest that while combination therapies are likely to work in the short term, parasites may develop resistance to multiple, non-structurally related anti-malarials over time. With regards to malaria control efforts, this indicates a need for careful monitoring, proactive policies, and a multi-focused approach that focuses on vectors, the human host, and environmental factors in malaria transmission along with continued efforts to find other anti-malarials.

Parasites that are resistant to QN have been reported in Thailand since the 1980s [15]. QN was used in combination with tetracycline, and remains to be used as a second line treatment for falciparum infection and as a monotherapy for infections in pregnant patients [25]. These data indicate general sensitivity in parasites with regards to QN, which is expected given its infrequent use in the region. Some studies have indicated shared resistance mechanisms between the amino-alcoholic drugs and QN [23,25]. As such, the extensive use of MQ both alone or in ACT suggests that continued monitoring of QN resistance is strongly warranted.

ART has been used for several decades in Cambodia, Vietnam and China, however its use is relatively new in other parts of the GMS. In the last several years there have been reports of potential reduced sensitivity in *P. falciparum* with regards to ART drugs in Thailand. The first reports concerned reduced *in vivo* sensitivity along the Thai-Cambodia border and more recently, tests of *in vitro* sensitivity along the Thai-Myanmar border have shown reduced sensitivity to ART and ART derivatives [5-7,22]. This research further supports these previous findings. While most of the sentinel provinces exhibit low ART IC50 values, Tak (on the Myanmar border) and Trat (along the Cambodian border) both show high, fluctuating values (Figure 3). It is curious to note that these data indicate high ART and MQ IC50 values in Tak and Trat Provinces around the same time that ATS-MQ became the standard therapy for falciparum malaria in the same locations. It is possible that sensitivity to ART was present because the drug was already in use by populations that frequently move across the borders to Myanmar (bordering Tak) and Cambodia (bordering Trat) where ART drugs has been in use for a longer period of time. However, since testing was done by different laboratory technicians, places, and times, and since there are no detailed data on individual assays it is possible that the high ART values in Tak and Trat represent variations in laboratory conditions. The data also indicate that DHA IC50 values are high in north-western Thailand and perhaps especially in Tak Province (Figure 4). These data are more reliable than the microtest III data, since they include results from individual assays and have error bars for comparison across regions. Given the importance of ART and its derivatives as the last line of defence against malaria, these findings are alarming. Careful monitoring and use of ART family drugs is crucial for global malaria control and eradication efforts.

There are several limitations to this study, which need to be considered for improvement in the future. The sampling method was not systematic, taken opportunistically from patients seeking treatment from malaria clinics and having high parasitaemia, and thus the samples may not represent the overall parasite population. Direct comparison of IC50 values across sentinel provinces may be problematic because of varying laboratory assay conditions and levels of technical training. Mixed infections, which are likely to be much more common in the GMS than was previously thought [19], can lead to inaccuracy in drug sensitivity testing [26]. In addition, while several studies have shown a rough correlation between *in vitro* tests and clinical outcomes, the potential remains for discrepancies between the two [27]. Nevertheless, given the limited resources (especially funding and skilled labour), *in vitro* tests remain an important proxy measure for clinical outcomes (e.g., parasitaemia quantification) in malaria infections. The HRP2 method is less labour intensive than the WHO microtest, meaning that it can be used in a more systematic way. If resources permit, it is ideal to test the same drugs, at least each year, continuously over time. Furthermore, quality control measures including keeping detailed records concerning variance in assays, changing laboratory personnel, and other laboratory conditions, should be implemented and preserved. Finally, combination of this test with more accurate *in vitro* tests of culture-adapted parasites and clinical efficacy (e.g., *in vivo* parasitaemia over time, after the administration of anti-malarials) data will significantly strengthen the drug surveillance data.

Furthermore, surveillance efforts in the GMS and Thailand must adapt to changing malaria epidemiology. Over the last several decades, *P. falciparum* prevalence has decreased markedly in Thailand, while the proportion of *P. vivax* cases has increased. In general, vivax malaria has largely been ignored in epidemiology, but researchers have come to the realization that the so-called ‘benign tertian malaria’ poses a credible threat to public health [28-30]. Yet, drug resistance surveillance for this parasite has remained scant [29]. Although CQ remained relative effective in treating *P. vivax* malaria in some regions of the GMS such as Thailand and China [31,32], cases of vivax resistance have been recorded the GMS [33-37]. In 2010 Cambodia began using a combination therapy (DHA-piperaquine and primaquine) as the first line treatment for *P. vivax* malaria. This points towards a demand for more close monitoring of *P. vivax* resistance to commonly used anti-malarials especially CQ and potentially the need for a combination therapy for *P. vivax* cases [38].

In the face of potential parasite resistance to ART and its derivatives, several actions should be taken. Since the border regions surrounding Thailand have historically been the site of persistent drug resistance (which may have subsequently spread to other parts of the world), surveillance of drug resistance in this region is crucial for global health and drug policy concerns. Currently there are *in vivo* studies of drug resistance, focusing on parasite density over time within patients that have been administered anti-malarials, in all nations of the GMS. However, such testing is troubled by the difficulty of following some patients over time and not all subregions or areas within the GMS have the manpower to conduct lengthy testing for drug resistance. The geographical range of both *in vitro* and *in vivo* surveillance should expand into areas of the GMS that aren’t covered by current surveillance efforts. A more holistic, co-ordinated approach would ensure better surveillance across the GMS, revealing a more comprehensive picture of the drug resistance situation. There should be a move towards the careful regulation of anti-malarials and combination therapy should be used throughout the region. Several studies have shown that fake and substandard pharmaceuticals are widespread throughout the GMS; therefore surveillance efforts should also be put into place to investigate this crucial problem. Finally, while anti-malarials are undoubtedly important for malaria control efforts, other modes of control should continue to be pursued. For example studies into behavioural components, environment and disease interactions, as well as both human and vector ecology have been and will continue to be important for malaria control efforts.

## Competing interests

The authors declare that they have no competing interests.

## Authors’ contributions

RL, WS, JP, and JS carried out laboratory procedures, including drawing blood samples and testing parasite sensitivity to anti-malarials. Surveillance was implemented and carried out by RL, WS, JP, and JS. DP, GY, JW, and LC collected the raw data, did the statistical analyses, and wrote the paper. All authors contributed to the interpretation of results, the conclusion, and approved the final manuscript.
